# A Case of Bursting of the Bladder, with Remarks

**Published:** 1888-04

**Authors:** Marcell Hartwig

**Affiliations:** Buffalo, N. Y.


### *A CASE OF BURSTING OF THE BLADDER, WITH REMARKS.*

By Dr. MARCELL HARTWIG, Buffalo, N. Y.

Bet., *æt. 40*, a strong man, was jammed in between two railroad cars on October 20, 1887. Both trochanters were caught, and, through the pressure, the man was rotated around the axis of his body, whereby his full bladder certainly suffered pressure. At the start, the right leg was very painful, while moved, in the hip-joint; ditto the right sacroiliac joint and the right trochanter. The left trochanter was hardly sensitive. Two inches above the symphysis pubis was a hardly noticeable red streak on the skin, an abrasion close above the right trochanter. The perineum was slightly sensitive, and from the orifice of the penis, a drop or two of blood were oozing. It soon appeared that the right hip was not seriously hurt, while the articulatio sacroiliaca was swelled, without dislocation. The interest of the case centered around the bladder. Since five o'clock in the evening till ten on the next morning, while using sixteen doses of ten drops chlorodyne each, he did not pass a drop of water. I then introduced a Nélaton catheter No. 22 F. without trouble, but only a few drops of blood came. Even attaching a suction-pump, only a couple of tablespoonfuls of blood appeared. The usual signs of peritonitis were wanting, the pain in the lower abdomen excepted. Percussion showed a dullness, of the form of a bladder, reaching till to the middle between symphysis and navel. The space was oval and felt somewhat more resistant. Thus, either the bladder was distended with blood, or urine was accumulated between bladder, peritonæum and frontal wall of the abdomen, imitating the shape of the bladder. I decided the latter condition to prevail, assuming that the suction would have sufficed to force blood-clots out and to draw urine, were there any in the bladder. I proposed to tap the dullness with Dieulafois' troicar. That was refused, and I had a chance to observe the forces of nature. Twenty-four hours later—that is, after forty-one hours—no urine yet. Silver catheter, well-disinfected, of course, introduced and moved a little to dislodge blood-clots. Nothing came but a teaspoonful of blood. Dullness reaches to the navel; shape of bladder. Next night, after at least fifty-three hours, he passed, repeatedly, urine, the dullness not diminishing. Even after eight days, *status quo*. The conclusion was sure that now, after no space existed in the prævesical space, the urine had to come the natural way. After eight days, he was able to stand and to pass a glass of urine at once. If he then sat down on the edge of the bed for five minutes, he could pass again a glassful, a nice proof that during that time urine had leaked from the prævesical space into the bladder, because kidneys cannot manufacture urine as fast as that. At the same time, the dullness lowered two fingers' width. On October 30th, he could pass no two portions any more. Dullness in the center between navel and symphysis. This makes us, of course, conclude that the rent in the bladder had closed. The first portion was every time more cloudy than the second, coinciding with the microscopical appearance of numerous resp.; few leucocytes. The dullness lost, by-and-by, the oval shape and widened in the lower region, especially towards the left. On October 30th, I noticed on the *left* trochanter a small sac, which seemed like a late burst of a subcutaneous vessel, but the distinct fluctuation roused already my suspicion to the question whether there was no urine in there. On November 1st, the bag was already considerable, and I emptied it with the hollow needle, obtaining a pint, almost, of clear urine.

The dullness reached, on the left side, along the whole length of Poupart's ligament, till to the spina anterior superior ilei; the upper edge stood midway between navel and symphysis pubis. Light uretritis. No erections. On November 3d, I emptied again a glassful of urine from the sac on the trochanter. A test on gelatine, taken with all precautions, remained sterile, as after some time appeared. On November 13th, the sac was emptied the last time. The urine appeared clear and not decomposed. On November 15th, suddenly, the right testicle swelled. Well-characterized epididymitis, lasting for six days. In the urine, which the man passed *per vias naturales*, there was sufficient pus, but I was not able to detect any gonococci, while the man denied of ever having had any gonorrhœa. The stream of urine is slightly thinned. This circumstance is probably explained by the fact that, right from the start, there was some hemorrhage in the perineum, the same having had a bluish hue. January 1, 1888, the man was well without further treatment.

*Epicrisis.*—Why the man had urethritis and epididymitis, I am not prepared to explain; ditto not why the urine accumulated just on the *left* trochanter while the man was always laying on the back. It is hard to make up a definite opinion, too, in regard to the question whether the urine diffused through the sciatic notch or under Poupart's ligament. The first presumption seems to me the more probable, because abscesses from the spinal column reaching Poupart's ligament pass underneath of it, and not unto the trochanter. Highly interesting is the condition of the subfascial urine; not a trace of decomposition, no uræmia, no micrococci. I am sorry of not having taken any notes of temperatures, but, as far as I can remember, the first four or five days the temperature was raised to about 103, thus an aseptic fever present, as seen likewise with large subcutaneous fractures.

Thus we see here one of the few cases where the *vis medicatrix naturæ* sufficed, with very little aid, to perfect a cure of ruptured bladder. It is almost strange that the peritonæum did not burst anywhere from so large an effusion; but if we consider that on the other side of that membrane an even pressure from the adjacent intestines exists, the fact appears less remarkable. As far as I had occasion to peruse the literature, I found the recommendation always repeated to make an incision above the symphysis pubis in subperitonæal rupture of the bladder. This case shows that the incision is not an absolute necessity. At all events, distinctions will have to be made, according to whether the bladder was previously in perfect order or perhaps affected with catarrh. (Urethral stricture was often a cause of rupture.) If the bladder is, at the time of the accident, in a diseased condition, sub (pro) peritonæal phlegmone would have to follow the rupture. With absolute antisepsis, an incision would certainly never do any harm. On the other hand, I would certainly prefer to leave things to nature, with a previously healthy bladder, where external conditions would prevent to maintain an absolute antisepsis, like here, where the patient's will prevented me from active treatment. Another case essentially analagous I could not find in the literature to which I have access here. One thing must exist for a happy result of the expectant plan, and that is, the rupture must be extra-peritonæal. This is the main question in regard to treatment, because intra-peritonæal ruptures left to themselves are invariably fatal. Here the urine fills the peritonæal cavity without finding a limit, and invariably produces peritonitis with uræmia. The differential diagnosis is the punctum saliens, and such is not by any means easy, because in intra-peritonæal rupture the symptoms were often, at the start, so insignificant that patients went long distances to see the doctor.*

---

* More about differential diagnosis: See diagnosis and treatment of intra-peritonæal wounds of the urinary bladder, by Alexander W. Stein, *Medical Record*, February 6, 1886, page 146. *Medical News*, February 5, 1887, page 159, and 1884, page 418. R. Weir, *Medical Record*, January 22, 1887, page 88. Lidell, John A., rupture of abdominal viscera. *American Medical Journal*, April, 1867, page 358 and following. Heath, Ch., *Lancet*, March 1, 1879. B. Beck, *Deutsch Zeitschcr., für Chir.*, Bd. xix., reported in *American Journal of Medical Science*, October, 1884, page 585.

What course, then, is the most reliable? I do decidedly believe, with reliable antisepsis, the probatory incision, which has to end in drainage, if an extra-peritonæal rupture is met with. If, again, intra-peritonæal rupture is found, the bladder is to be stitched with two rows of stitches, without inclusion of the mucosa, the peritonæal cavity is to be cleansed with antiseptics (I prefer ⅓ per cent. salicylic acid solution), and the caveum Douglasii drained.

Concerning drainage of Douglas's pouch after suture of the bladder, I am convinced that it gives greater security than the simple sewing of the abdominal incision, though it exposes to infection after the operation. Here I stand entirely on the lamented Sims' shoulders, believing that the future will return more and more to drainage in all abdominal operations. Where or how shall we know if the cavity of the abdomen was soiled *already* before an operation, whether each coccus is dead or removed? Even if experience teaches that a good many may be harmlessly absorbed, the drainage remains the grandest safety-valve of a wound; the micrococci certainly more harmless when flowing out as though absorbed. The execution only of the drainage is less convenient in man than in woman. To drain upwards in the recumbent position, is not the most suitable way, and downwards, through the *perineum*, I am not aware that somebody has tried it. All the same, I do believe the future may show this way practicable. Once I have experimented in this respect on the cadaver. It is not easy to pass between bladder and rectum without pricking either. I had tried a drain of the thickness of a little finger. With a thinner one, it will be less difficult. At any rate, the rectum should not be pierced; rather let us chafe the prostate. Already the gas from the rectum entering a drain would induce fatal septicæmic peritonitis. In passing the drain, the left index ought to be in the rectum, the thumb on the perineum, and, for piercing the latter, an instrument should be used in Douglas's pouch, analagous to the one which is used in our kitchens to draw the slices of bacon through the filet.

Longer than five days a drain, generally, need not lay, and that long the stools can be retarded and asepsis insured. I am pretty sure a simple drainage of this kind might cure a number of intestinal perforations without using any further means.

For sewing I should use juniperized or chromacized alcoholic catgut. Some have pleaded for silk, and even used it with success, but I should fear that silk would finally fall into the bladder and become a nucleus of stone. Peritonæal ruptures which have been cured by other means than sewing are only a very small number in the literature—I almost think only three—while extra-peritonæal ruptures cured without incision seem not to be much more numerous. The correct conclusion, therefore, is the above given. The differential diagnosis never being absolutely certain, what is the use losing time with catheterism, etc., while it is a fact that, with intra-peritonæal rupture, every hour lost jeopardizes the result more and more? This loss of time counts more than anything else. This is plainly seen in the unfailing good results where the bladder was accidentally cut during an ovariotomy or other abdominal operation, and sewed immediately. I never read a case where the result was fatal. The number of successful sewings of the ruptured bladder, though only amounting to half a dozen, shows they were all early cases. Of course, a test has to be made, after stitching, whether the bladder is fluid-tight. Only the question whether a catheter is to be kept in, or whether sufficiently frequent catheterizing has to follow, is dubitable in my mind, though I am inclined to prefer the latter; if not the lateral lithotomy, which was used often enough without sewing, to a certain advantage, while the stitched successful cases of the literature got along without the prostatic incision. Were the patients not shocked enough through the accident, this incision could not count much. In subjects we have to deal with, the incision is an additional risk, and well worth considering. Only the shots through the bladder seem to deserve different treatment. Here the experience has shown remarkably frequent cures, notwithstanding that often terrible complications exist. So Cheselden, Hildanus and Garengeot already have operated stones where bullets formed the centers, showing cured cases of shot bladder. The easy discharge of the urine through the channel of the bullet explains the relative frequency of cured shots of the bladder. A good many reported cases seem to have been extra-peritonæal. The first care in open wounds of the bladder will be to keep them open, so that the urine will ooze out perfectly easy, until sufficient adhesions are formed, preventing escape into the peritonæal cavity or into connective tissue. If possible, a drainage-tube should be introduced through the wound, but if any boring, etc., should be required, it would be far preferable to make lateral lithotomy for drainage, a proceeding which was frequently successful in those cases. Of late, it *was proposed* to connect the permanent catheter with continual siphonage into an antiseptic fluid on the bedside. It appears well on paper, but such long presence of a catheter is badly borne, causing violent contraction of the bladder, and all motions of the patient are liable to spoil the beautiful idea in a real case, though the possibility of a successful use is undeniable.

*Enfin.*, the danger of penetrating gunshot wounds of the bladder will be reduced yet by modern antisepsis, but the best hopes are in store for future intra-peritonæal bursts treated by early sewing. MacCormack had already two successful cases: for example, one with abdominal drainage and catheter in the bladder, one without both.

Six per cent. is the number of recoveries, according to St. Smith, in wounds of the bladder. Let us hurry, in future, to do the probatory incision, and not be induced by a happy stray case, like the one related, to temporize, and the percentage of recoveries will steadily increase, while it will become less and less true, what almost held its own through ages, that what Hippocrates says: "That every case is doomed."

